# Toxic Trace Elements in Meat and Meat Products Across Asia: A Comprehensive Literature Review and Implications for Human Health

**DOI:** 10.3390/foods14010009

**Published:** 2024-12-24

**Authors:** Jose L. Domingo

**Affiliations:** Laboratory of Toxicology and Environmental Health, School of Medicine, Universitat Rovira i Virgili, Sant Llorens 21, 43201 Reus, Catalonia, Spain; joseluis.domingo@urv.cat

**Keywords:** arsenic, cadmium, mercury, lead, meat and meat products, human health risks, Asian countries

## Abstract

Meat and meat products are vital sources of essential nutrients for human health and development. However, an excessive or inappropriate consumption can pose significant health risks. In 2015, the International Agency for Research on Cancer (IARC) classified red meat as “probably carcinogenic to humans” and processed meat as “carcinogenic to humans”, yet the role of environmental contaminants in these products was not addressed. The present review focuses on human exposure to toxic trace elements (arsenic, cadmium, mercury, and lead) through meat and meat products in Asia, covering scientific literature from 1 January 2000, to 30 August 2024. Based on the citations in PubMed and Scopus databases, Asia is the region with the highest number of reported studies, with China contributing the most data. Concentrations of toxic elements in meat vary significantly depending on animal species, specific tissues consumed, and geographic origin. Correspondingly, estimated daily intakes of toxic elements from meat consumption also differ across studies. While some research highlights negligible carcinogenic and non-carcinogenic risks, others indicate potential health concerns due to elevated toxic element exposure in specific cases. However, similar to observations with organic pollutants, meat and meat products in Asia are not among the primary dietary sources of exposure to toxic elements for humans.

## 1. Introduction

For centuries, and even millennia, the consumption of meat and meat products has been, and continues to be, widespread across almost the entire world. Meat is a vital source of nutrients essential for human development and health. However, inadequate or excessive consumption may lead to issues related to health, the environment, and society [1,2,3]. Regarding human health, the balance between the benefits and risks of meat consumption varies across regions, countries, cultures, and dietary habits. Among the benefits, to be a nutrient-rich source is probably one of the most relevant, given its status as a nutrient-dense food source particularly significant. Meat provides high-quality proteins, essential fatty acids, various vitamins, and trace elements. Additionally, it is energy-dense and satiating, making it a crucial food source for populations with limited food security. In developing regions, meat consumption can combat malnutrition and provide vital energy. Nevertheless, an excessive meat consumption is associated with adverse health effects, such as cardiovascular diseases linked to high intakes of red and processed meats, while obesity, type 2 diabetes, and even antibiotic resistance (due to the use of antibiotics in livestock production) are also other potential health risks [4,5,6]. Furthermore, in 2015, the International Agency for Research on Cancer (IARC) classified red meat as *“probably carcinogenic to humans” (Group 2A)* and consumption of processed meat as *“carcinogenic to humans” (Group 1).* Since then, the consumption of red and processed meats has been linked to an increased risk of colorectal, prostate, and breast cancers [7,8,9,10,11]. Recent studies have also investigated the role of chemical contaminants in the carcinogenicity associated with regular consumption of red meat and meat products [12,13,14]. It was concluded that meat and meat products are not major contributors to the dietary intake of carcinogenic organic contaminants, such as dibenzo-*p*-dioxins and dibenzofurans (PCDD/Fs), polychlorinated biphenyls (PCBs), benzo[a]pyrene and other PAHs, and hexachlorobenzene (HCB) [12]. However, it is worth noting that the review conducted by Domingo [12] focused exclusively on organic contaminants.

Given the above, the objective of this study was to review research carried out in Asian countries during the 21st century on the concentrations of As, Cd, Hg, and Pb (as well as Cr and Ni) in meat and meat products. When data were available, the health risks posed by these elements to consumers were also reviewed. The scientific literature reveals that Asia, particularly China, has generated the largest volume of research on this topic since 2000. To ensure conciseness and avoid complexities in comparing data across continents, this review is limited to studies from Asian countries. The interest in health risks related to dietary exposure to chemical contaminants, including those found in meat, remains significant. Consequently, the present article focuses on recent studies examining human exposure to toxic metals and metalloids through the consumption of meat and meat products. While some data on non-essential elements have also been reviewed, the focus is on four toxic elements: arsenic (As), cadmium (Cd), mercury (Hg), and lead (Pb). Arsenic and Cd are classified as potential carcinogens by the IARC [15], while Pb is considered a probable carcinogen by the United States Environmental Protection Agency (US EPA). Other metals such as beryllium (Be), chromium (Cr-VI), and nickel (Ni), are also potential carcinogens [16]. Notwithstanding, Be is rarely detected in meats, and the interest in Cr (as Cr-III) and Ni pertains mainly to their essentiality rather than their carcinogenicity. Consequently, the purpose of the current paper was to summarize the available literature on the topic here reviewed. It is a literature review, not a systematic review or meta-analysis, as their methodologies are substantially different.

## 2. Search Strategy

The Scopus (https://www.scopus.com, accessed on 15 August 2024) and PubMed (https://www.ncbi.nlm.nih.gov/pubmed/, accessed on 15 August 2024) databases were used to search for published information related to the main topic of this review. The search period covered the timeframe from 1 January 2000, to 30 August 2024. The following keywords and their combinations were utilized: *meat*, *meat products*, *human exposure*, *human dietary intake*, *metals*, *metalloids*, *carcinogenic risks*, and *non-carcinogenic risks*. In addition to the articles here discussed, the search conducted in the databases used for this review also detected a series of citations that were either deemed irrelevant (“noise”), or did not align with the purpose of the review, being therefore excluded. Only articles published in English were included.

## 3. Concentrations of Toxic Elements in Meat and Meat Products

The most relevant findings from scientific studies conducted in Asian countries, which are available in the databases (PubMed and Scopus) used to prepare this Review are summarized below. Due to significant differences in specific objectives and methodologies (e.g., analyzed elements, animal species, sample types, sampling years, dietary habits, etc.), the results of the reviewed studies are presented by country and year of publication.

### 3.1. China

Chen et al. [17] evaluated the daily intake of cadmium (Cd), lead (Pb), mercury (Hg), and arsenic (As) by the population of Xiamen (2005–2009) through various food groups, including meats (chicken, pork, and offal). The mean concentrations (μg/g) in chicken were Cd: 0.003, Pb: 0.008, Hg: 0.005, and As: 0.020. In pork, these concentrations were Cd: 0.002, Pb: 0.011, Hg: 0.004, and As: 0.023. In offal, the values were Cd: 0.055, Pb: 0.146, Hg: 0.002, and As: 0.005. The estimated daily intake (EDI) of these elements through meat consumption was highest for pork. Jiang et al. [18] analyzed 16 rare earth elements (REEs) in commonly consumed foods, including pork, pig kidney, and liver. Only a few REEs (Ce, Dy, Y, La, and Nd) were detected at relatively high levels, with mean and median concentrations in meat samples of 0.080 and 0.016 μg/g, respectively. In Hong Kong, Chen et al. [16] conducted a total diet study (TDS) to estimate exposure to metals like Al, Sb, Cd, Pb, MeHg, Ni, Sn, and V. The dietary exposure levels for Cd, Pb, and MeHg were below health-based guidance values (HBGVs). Although meat did not significantly contribute to the intake of these elements, MeHg exposure during pregnancy was noted as a potential public health concern due to risks to fetal health. Tang et al. [19] evaluated health risks from dietary exposure to Cd, Pb, and Hg in Zheijang. While marine products showed the highest levels of Cd and Hg, pork exhibited the highest mean concentration of Pb among the analyzed foods. Jin et al. [20] reviewed Pb concentrations in food between 2006 and 2012. They reported mean Pb levels of 0.074 μg/g in meat and 0.181 μg/g in offal, with dietary intake of Pb from meat and offal contributing 5.469 μg/day and 0.851 μg/day, respectively. Wu et al. [21] analyzed 14 elements, including Cd, Hg, Pb, and As, in meats and aquatic products from Shenzhen. They found the highest concentrations of As and Cd in aquatic products and animal viscera. The target hazard quotient (THQ) for aquatic products was significantly higher compared to meat products, with cumulative THQ values exceeding 1 when considering all meat products analyzed.

Cheng et al. [22] measured the levels of eight elements (As, Cd, Co, Cr, Cu, Hg, Pb, and Sb) in samples of vegetables, fish, and meat purchased in 2014 from markets in Huainan. Regarding the mean concentrations of the most toxic elements in meat samples, the highest As levels were detected in chicken (0.036 μg/g), followed by beef (0.029 μg/g), while the highest Cd levels were found in duck (2.61 μg/g) and chicken (1.09 μg/g). The highest mean Hg levels were found in duck (0.790 μg/g) and chicken (0.694 μg/g), while Pb levels were highest in beef (0.165 μg/g) and pork (0.100 μg/g). The EDIs for the four toxic metals through meat consumption in Huainan were 0.041, 0.001, 0.001, and 0.176 μg/kg bw/day for As, Cd, Hg, and Pb, respectively. On the other hand, Zhang et al. [23], using data from the Chinese National Food Contamination Monitoring Program between 2001 and 2009, assessed the risks of Cd through long-term dietary intake for children in Jiangsu Province. Meats were one of the food categories included in this study. The following Cd concentrations were found in kidney (pig, sheep), 1711 μg/kg, in liver (pig, sheep), 68.3 μg/kg, in meat (from mammals other than pig), 3.7 μg/kg, in pig meat, 12.6 μg/kg, and in poultry, 11.8 μg/kg. For children (2–17 years old), the percentage contribution of pig meat to the total EDI of Cd ranged from 4.4% to 4.6% In another survey, Yin et al. [24] measured the total Hg (THg) and methylmercury (MeHg) content in poultry (chickens, ducks, and geese) from two farms near the Wanshan mercury mine in Guizhou Province, southwestern China. The muscles (leg and breast), organs (intestine, heart, stomach, and liver), and blood of the three species were collected in 2015. The highest Hg levels were found in the liver (THg: 23.2–3917.1 μg/kg; MeHg: 7.1–62.8 μg/kg), followed by the blood (THg: 12.3–338.0 μg/kg; MeHg: 1.4–17.6 μg/kg). In chickens, the estimated Hg burdens were 15.3–238.1 μg for THg and 2.2–15.6 μg for MeHg, while in ducks, they were THg: 15.3–238.1 μg and MeHg: 3.5–14.7 μg, and in geese, THg: 83.8–93.4 μg and MeHg: 15.4–29.7 μg.

During the 2013–2014 period, Hu et al. [25] collected samples of fresh chicken products from markets in Guangdong province to measure various metals/metalloids (As, Cd, Co, Cr, Cu, Mn, Ni, Pb, Se, and Zn) and evaluate the human health risks associated with these elements. Breast, drumstick, gizzard, heart, kidney, and liver tissues were specifically analyzed. For the toxic elements (As, Cd, and Pb), the highest and lowest concentrations were as follows: As, 0.049 μg/g (liver) and 0.012 μg/g (drumstick); Cd, 0.019 μg/g (liver) and 0.001 μg/g (drumstick); and Pb, 0.116 μg/g (breast) and 0.042 μg/g (kidney). The study concluded that exposure to inorganic As (at elevated levels) from chicken consumption could pose significant health risks to consumers. In another survey conducted by Wang et al. [26], the concentrations of 11 elements (Mn, Zn, Cu, Sr, Cr, Ni, As, Cd, Pb, Co, and Sb) were determined in 29 food types, including pork, beef, and poultry. The samples were collected in 2014 in Chengdu, China. The concentrations of As, Cd, and Pb (in μg/kg dry weight) were as follows: in pork samples, As, 41.8; Cd, 2.06; Pb, 30.4; in beef samples, As, 44.9; Cd, 2.17; Pb, 32.6; and in poultry samples, As, 30.2; Cd, 2.79; Pb, 31.0. These results indicated that some meats had As, Cd, and Cr levels above the respective limit values. In a similar study conducted in Beijing, Liang et al. [27] measured the concentrations of Cr, Cd, Pb, As, and Hg in various foodstuffs, including mutton, beef, chicken, and pork. The mean concentrations were as follows: mutton, 0.654, 0.010, 0.128, 0.046, and 0.029 μg/g; beef, 0.504, 0.015, 0.201, 0.077, and 0.010 μg/g; chicken, 0.650, 0.031, 0.291, 0.045, and 0.017 μg/g; and pork, 0.483, 0.003, 0.029, 0.043, and 0.015 μg/g. The average concentrations for these five elements were 0.573, 0.015, 0.167, 0.053, and 0.018 μg/g, respectively. The intake of these elements through meat consumption was 1.5, 60.1, 17.5, 5.5, and 1.9 μg/day for Cd, Cr, Pb, As, and Hg, respectively, versus total EDIs of 29.5, 205.5, 95.2, 34.0, and 15.6 μg/day for the same elements. The highest metal contribution to the respective EDIs came from Cr, with a percentage of 29.2% of the total intake of this metal.

The dietary intake of lead (Pb) in China has been specifically assessed in various studies conducted within the country. Liu et al. [28] examined the regional characteristics of dietary Pb exposure in the Chinese population, while also assessing the potential health risks across different regions due to Pb intake through various foodstuffs. Data were obtained from total diet studies (TDS) conducted in the country, with the most recent data (from the 5th TDS) collected between 2009 and 2013. Regarding meat and meat products, the highest intakes in the 5th TDS were found for barbecued pork, duck, pig liver, and pork skin aspic. It was evident that there were significant variations in Pb concentrations, as well as in the dietary intake of this toxic element, not only in these meat products but across different food groups and items assessed. In another study related to the risks of dietary Pb intake, Zhang et al. [29] estimated Pb exposure through food for residents near the mining areas of Nandan County. Food samples were collected from 24, 27, and 25 households located near three mining areas in 2013 and 2014. The most consumed meats in the region—pork, chicken, and duck—were included in the study. The results showed that Pb levels in meat and meat products were notably significant. The mean Pb concentration in meat was 3.22 μg/g (range: 2.75–4.04 μg/g), and the contribution of meat to the total Pb intake (1400 μg/day) was 381.8 μg/day, surpassed only by the contribution from vegetables (716.7 μg/day). The authors recommended that residents increase their consumption of fish and grains instead of meat. Using data from the 5th TDS, Wei and Cen [30] assessed dietary cadmium (Cd) exposure for residents in the Heilongjiang, Jilin, and Liaoning provinces in northeastern China. Meats and meat products assessed included pork, chicken breast, pig liver, sausage, roasted chicken, beef, pork skin aspic, lamb, dorking, braised spareribs, chicken liver, roasted duck, and coagulated pig blood. There were significant differences in meat consumption across the three provinces: 48.61, 114.57, and 93.18 g/day, respectively. Accordingly, notable differences in Cd intake through meat consumption were expected, with margins of safety (MOS) of 4.55, 1.82, and 2.85 for the three provinces. Between 2017 and 2019, Zhang et al. [31] evaluated the benefits and risks of dietary selenium (Se) and its associated metals in 10 regions of China. They found that Se-rich agro-foods could serve as an effective supplement compared to common agro-foods. Meats were the main dietary contributors of Se, with Se levels in meat ranging from 0.1728–0.2465 μg/g (wet weight). The associated metals in meat samples were found to range as follows: Pb (0.0302–0.0352 μg/g), arsenic (As) (0.1121–0.1195 μg/g), Cd (0.0010–0.0010 μg/g), mercury (Hg) (0.0064–0.1140 μg/g), and chromium (Cr) (0.1018–1.0380 μg/g). A negative outcome of the study was that Cr in Se-rich agro-foods might pose health risks for individuals under 18 years old, though the study did not specify which meats were analyzed.

Pei et al. [32] conducted a study to determine the levels of Cr, As, Cd, Hg, and Pb in pork samples. The samples included muscle (shoulder, ham, loin, tenderloin, and belly) and edible offal (liver, kidney, and intestine) from pork purchased in Nanjing. The results showed that the daily intake of the analyzed elements (Cr, As, Cd, Hg, and Pb) was clearly below the recommended values, with intakes of 3.00, 2.14, 1.00, 0.57, and 3.57 μg/kg body weight/day, respectively. Moreover, the target hazard quotients (HQ) and total target hazard quotients (THQ) were all below 1.0, indicating that the usual consumption of muscles and edible offal from pork would be safe for consumers. Similarly, Wang et al. [33] measured Pb, Cd, Hg, and As levels in samples from 13 food groups purchased in Shenzhen markets. One of the groups analyzed was meat, though specific types were not reported. The mean levels of Pb, Cd, Hg, and As in the meat samples were 0.044, 0.0237, 0.0056, and 0.021 μg/g, respectively. Meat was one of the four major food contributors to the cumulative hazard quotient (HQ) and hazard index (HI) values for Pb, Cd, Hg, and As. Han et al. [34] measured the concentrations of As, Cd, Cr, Cu, Hg, Ni, and Pb in fresh meat samples (pork, beef, mutton, chicken, and duck) purchased from markets in the Zhejiang province. The mean concentrations of these elements were 0.018, 0.002, 0.061, 0.801, 0.0038, 0.055, and 0.029 μg/g, respectively. Some samples exceeded the maximum allowable concentrations (MACs), with one sample for As, two for Hg, and ten for Pb. In China, the MACs for As, Cd, Cr, Hg, and Pb in meat are 0.5, 0.1, 1, 0.05, and 0.2 μg/g, respectively. The exposure assessment indicated relatively low health risks for individuals consuming the analyzed meat products, with only small percentages of samples exceeding the MACs (0.09% for As, 0.19% for Hg, and 0.94% for Pb). Another recent study on this topic was conducted by Wang et al. [35], who measured concentrations of various elements (Al, As, Cr, Cd, Cu, Ni, Pb, and Zn) in samples of foodstuffs (and drinking water) widely consumed in industrial regions of northern Ningxia. Meat samples included pork, beef, and mutton. On average, the concentrations (μg/g) of the most potentially toxic elements were as follows: As, 0.218; Cd, 0.000; Cr, 0.472; Ni, 0.000; and Pb, 0.078. The non-carcinogenic risks (measured by the Hazard Index, HI) for meat consumers were: 0.0259 (Al), 0.4080 (As), 0.1012 (Cr), 0.000 (Cd), 0.0187 (Cu), 0.000 (Ni), 0.0136 (Pb), and 0.0995 (Zn), with the global HI (<1) being 0.7269. The carcinogenic risk associated with meat consumption was 2.11 × 10^−4^, a value higher than those for the other food groups (fruits, vegetables, tubers, beans) included in the survey, and was only exceeded by the carcinogenic risk from drinking water (2.34 × 10^−4^).

The results of studies on human exposure to metals/metalloids through meat consumption conducted in China and published in 2024 are summarized below. Han et al. [36] reported the Ni concentrations in samples of various food items from several food groups collected in the Zhejiang Province. The meat and meat products group included sausages, Chinese bacon, and other (not specified) meat products. The median concentrations of Ni were 0.262 μg/g for sausages, 0.252 μg/g for Chinese bacon, and 0.116 μg/g for the other meat products, with a median level of 0.258 μg/g for all analyzed meat samples. Overall, dietary exposure to Ni was at acceptable levels, except for the age group between 0 and 6 years, where the estimated daily intake (EDI) was found to be the highest. For this group, the consumption of Ni-contaminated foods exceeded the recommended tolerable daily intake (TDI) of 13 μg/kg body weight per day. However, the exposure to Ni from meat consumption was relatively low compared to other food groups. Recently, Qin et al. [37] determined the concentrations of Cu, Cr, V, Ni, As, Se, Sn, Cd, Pb, Sb, Mn, Ba, and Hg in meat and meat products collected from various cities in Shandong Province. The samples included livestock meat and offal, as well as poultry meat and offal. For the 13 analyzed elements, the total mean concentrations were 1.56 μg/g for meat and 39.8 μg/g for offal. Regarding the potentially most toxic elements, Cr, Ni, and Pb were detected in 100% of the analyzed samples, while Hg was found in 95%, Cd in 51.3%, and As in 21.3%. The total HI and Hazard Quotient (HQ) values indicated potential health risks for individuals consuming large quantities of meat and meat products. On the other hand, Zeng et al. [38] investigated the relationship between the levels of various metals (Cr, Cd, Cu, Zn, Ni, and Pb) in the hair of residents in Chengdu and their exposure to these same metals through the diet. Among the foodstuffs measured for these elements, the study included meats such as pork, beef, and chicken, as well as pig offal (gizzard, heart, intestine, liver, kidney, tripe, and brain), along with duck intestines and aorta, and bovine rumen and omasum. The highest concentrations of Cr, Cd, Cu, Ni, and Pb were found in cereals, while Zn was the only trace metal with the highest concentration in meat and meat products. Cereals, followed by meats, were the main contributors to the dietary intake of the analyzed metals and, consequently, to the concentrations detected in the hair of the population in the area. A summary of the studies conducted in China is shown in Table 1, and the studies conducted in Asian countries (excluding China) is shown in Table 2.

### 3.2. Bangladesh

Ullah et al. [39] determined the levels of several elements (Pb, Cd, Cr, As, Hg, Mn, Fe, and Zn) in three varieties of chicken meat (broiler, local, and Sonali) purchased from markets in Dhaka. The dietary intake of these elements was subsequently estimated, and both carcinogenic and non-carcinogenic risks for consumers were evaluated. The concentrations (μg/g) of the analyzed elements ranged as follows: 0.03–2.73 for Pb, 0.01–0.015 for Cd, 0.025–0.67 for Cr, 0.04–0.06 for As, 0.01–0.015 for Hg, 0.15–0.63 for Mn, 2.50–38.6 for Fe, and 1.02–19.4 for Zn. The MACs were exceeded only by Pb. However, the target hazard quotient (THQ) and total target hazard quotient (TTHQ) for the combined elements, as well as the carcinogenic risks for lifetime exposure, were below the respective allowable benchmarks. In a separate study also conducted in Dhaka, Hossain et al. [40] measured the concentrations of Pb, Cd, Fe, Cr, Cu, and Zn in various chicken tissues (muscle, liver, gizzard, heart, kidney, and brain) regularly consumed by the Bangladeshi population. They also assessed the non-carcinogenic and carcinogenic risks. The levels (μg/g) in different chicken parts ranged as follows: 0.33 ± 0.2–4.6 ± 0.4 for Pb, 0.004 ± 0.0–0.125 ± 0.2 for Cd, 0.006 ± 0.0–0.94 ± 0.4 for Cr, 4.05 ± 4.2–92.31 ± 48.8 for Fe, 0.67 ± 0.006–4.15 ± 2.7 for Cu, and 4.45 ± 0.62–23.75 ± 4.3 for Zn. The THQ and TTHQ values were found to be < 1 (no carcinogenic risks for consumers), while the carcinogenic risks for elements with potential carcinogenicity were within acceptable limits. Bokthiar et al. [41] also measured the levels of As, Cd, and Pb in broiler chicken (bones, meat, and combinations of liver, kidney, and gizzard) purchased from farms and supermarkets in Dhaka, Chattogram, Gazipur, Rajshahi, and Barisal districts. While the concentrations of As and Cr were below the respective maximum residue limits (MRL), Pb levels exceeded the MRL. In a similar study, Chowdhury and Alam [42] measured the concentrations of six metals (Cd, Cr, Pb, Ni, Fe, and Cu) in meat, brain, and liver samples of seven animal species (poultry, Somali chicken, cow, quail, duck, pigeon, and goat) from Noakhali district. The non-carcinogenic and carcinogenic risks were also assessed. Considerable differences in metal levels were observed depending on the specific element, animal species, and tissue. For the two most toxic metals analyzed (Cd and Pb), the maximum and minimum values (μg/g) were as follows: for Cd, 0.46 (cow brain) and 0.04 (quail brain), 0.45 (cow muscle) and ND (quail muscle), and 2.40 (cow liver) and 0.69 (pigeon liver). For Pb, 7.54 (poultry brain) and 3.07 (quail brain), 5.01 (pigeon muscle) and 1.64 (quail muscle), and 81.87 (goat liver) and 2.79 (pigeon liver). It was concluded that overexposure to Cd from meat consumption could increase cancer risks, with children being more susceptible than adults.

### 3.3. India

In 2017, Mathaiyan et al. [43] collected samples from various parts (breast, liver, neck, and kidney) of common broiler chickens in five major metropolitan cities of Tamil Nadu. The levels of toxic elements As, Cd, Hg, and Pb were measured. The maximum and minimum mean concentrations (μg/g) of these elements were as follows: As, 0.78 (liver) and ND (neck and kidneys); Cd, 0.019 (breast and liver) and 0.014 (neck); Hg, 0.27 (breast) and ND (neck and kidneys); and Pb, 1.29 (breast) and 0.077 (neck). The estimated daily intake (EDI) ranges were 0.0002–0.0036 mg/day/person for As, 0.0002–0.0002 mg/day/person for Cd, 0.0008–0.0051 mg/day/person for Hg, and 0.0072–0.0182 mg/day/person for Pb. The authors concluded that, although the levels of these elements exceeded the maximum residue levels (MRL) in some meat and edible organs of chicken, the exposure to As, Cd, Hg, and Pb from chicken consumption in Tamil Nadu remained within acceptable limits. Similarly, Pappuswamy et al. [44] measured the levels of Cd, Pb, and Zn in samples of blood, intestine, breast, liver, and gizzard from chickens purchased in markets across three districts of Tamil Nadu. The maximum and mean concentration ranges (μg/g) for Cd and Pb were found in the intestine (0.26–0.42) and gizzard (ND-0.54) for Cd, and in the heart (2.9–6.24) and blood (ND-2.56) for Pb. The study concluded that, while the levels of these metals were generally within permissible limits, liver samples from chickens in Coimbatore exceeded these limits. Blood samples from chickens in all three districts could pose a potential risk to consumers.

### 3.4. Thailand

Nookabkaew et al. [45] measured the concentrations of As and Cd in samples of rice, eggs, vegetables, fruits, and animal meats, as well as infant formulas and drinking water. These samples were randomly collected from commercial centers in Bangkok between 2005 and 2008. High concentrations of both elements were found in pig kidneys and livers. The study indicated that exposure to As and Cd from consuming poultry or pig (liver or kidney) could potentially lead to a high weekly or monthly intake of these toxic elements, particularly for children. In a subsequent study, Aendo et al. [46] measured the concentrations of Pb, Cd, Co, and Cr in duck meat and duck eggs, assessing both non-carcinogenic and carcinogenic risks. Ninety samples of duck meat were obtained from 11 farms across various regions of Thailand. The mean concentrations in duck meat were 1.94, 0.22, 0.10, and 0.37 μg/g for Pb, Cd, Co, and Cr, respectively. Seventy-two percent of the Cd levels and 66.7% of the Pb levels exceeded the standards set by FAO/WHO. However, using the database of contamination in duck eggs and meat, the Total Target Hazard Quotient (TTHQ) for Pb, Cd, Co, and Cr was below 1 for all age and gender groups. The authors suggested that consuming duck eggs might pose a higher health risk than consuming duck meat.

### 3.5. Iran

Abedi et al. [47] analyzed the levels of Cd and Pb in five brands of cooked beef sausages (German, cocktail, hot dog, Lyoner, dry, and jambon) purchased in 2009 from supermarkets in Tehran. The results varied greatly depending on the type and brand of sausage. The maximum and minimum values for Pb were 158.7 μg/g ww (German) and 24.0 μg/g ww (cocktail), while for Cd, the values ranged from 13.5 μg/g ww (hot dog) to 2.2 μg/g ww (German). The mean levels of Pb and Cd across all samples were 53.5 μg/g and 5.7 μg/g, respectively. The EDI of Pb and Cd were 1.47 and 0.16 μg/day, respectively, both considered acceptable for consumers. Raeeszadeh et al. [48] also studied the levels of As, Se, and several other metals (Pb, Cd, Zn, Ni, Cu, Cr, and Co) in various meat samples (beef, turkey, sheep, and ostrich) collected in Sanandaj City, Kurdistan province. The highest and lowest mean concentrations (μg/g) for the most toxic were as follows: Pb (11.79 in sheep and 0.13 in turkey), Cd (4.31 in beef and 0.16 in ostrich), and As (0.88 in turkey and 0.20 in beef). The Target Hazard Quotients (THQ) for Pb ranged from 0.0069 (ostrich) to 0.769 (beef), for Cd from 0.121 (ostrich) to 3.283 (beef), and for As from 0.057 (turkey) to 0.393 (beef). A THQ value greater than 1 for Cd indicated that the levels in beef (and sheep) were at a warning level. Despite this, the concentrations of these metals, including As, Cd, and Pb, were generally considered acceptable based on the target risks for cancer. A review by Sarlak et al. [58] examined studies published during the 2010s on Pb concentrations in various animal-based food groups in Iran, including red and white meats and meat products. Few studies on metal concentrations in meats and meat products were identified, and the authors noted a decline in meat consumption in recent years in Iran, making it difficult to estimate exposure to metals through meat. A common finding was that Pb concentrations were higher in offal than in muscle tissues.

In turn, Hashempour–Baltork et al. [59] conducted a comprehensive review of toxic element levels, specifically As and Hg, in Iranian foods over the preceding two decades. The review focused on various food groups, including rice, fruits, vegetables, fish, seafood, drinking water, tea, and other miscellaneous items. Unfortunately, it excluded meat and meat products, the main objective of the present review. In contrast, more recently, Salim et al. [60], another group of Iranian researchers, published a systematic review investigating concentrations of the toxic elements As, Cd, Hg, and Pb in red meat samples, providing a critical perspective on heavy metal contamination in this important food group. Unlike the studies discussed in the current review, Salim et al. [60] analyzed data from meat samples collected globally, rather than exclusively from Asian countries. The authors identified geographical location as a primary factor influencing variations in the mean levels of toxic elements, with Asia and Africa exhibiting the highest concentrations. Other factors, such as the type of meat and fat content, were also found to impact health risks. The study concluded that, while the levels of Hg and As in red meat samples were generally below harmful thresholds for consumers, the elevated concentrations of Pb and Cd posed significant potential health risks associated with meat consumption.

### 3.6. Republic of Korea (South Korea)

Lee et al. [49] measured dietary exposure to As, Cd, Pb, and Hg in the South Korean population. A total of 116 foods/dishes (in a table-ready state), representing 66.5% of the total food intake, were included in this survey. The EDIs were: 38.5, 14.3, 24.4, and 1.61 μg/person (based on a 55 kg body weight) for As, Cd, Pb, and Hg, respectively. All values were within safe limits (under 30% of the respective Provisional Tolerable Weekly Intakes, PTWIs). The group of meat and meat products was not a significant contributor to the intake of these elements. For informational purposes only, in contrast to meat and meat products, other foodstuffs such as rice, vegetables, and fish and seafood made substantial contributions. Regarding Cd, the results from Lee et al. [49] align with those of Moon [61], who conducted a review on the relationship between Cd concentrations in blood and dietary intake among the South Korean population. It was found that fish and shellfish (3.00 μg/day), grains and cereals (3.36 μg/day), and vegetables (2.11 μg/day) were the primary contributors to Cd intake, after seaweed (3.66 μg/day). In that review, the mean total dietary intake of Cd was estimated to be 15.25 (range: 2.48–90.5) μg/day for the South Korean population.

### 3.7. Saudi Arabia

In 2010, Othman [50] measured Pb concentrations in various foods available in local markets in Riyadh. Chicken was included in the study. Human Pb exposure was estimated using a questionnaire (Food Consumption Survey) completed by the local population. Among the 10 food groups (vegetables, fruits, milk and dairy products, etc.) examined in that survey, the highest concentrations of Pb were found in sweets, vegetables, and legumes. In samples of chicken, the mean concentration was 0.026 ± 0.018 μg/g. However, considering the daily consumption of food items within their respective food groups, the highest dietary contributors to Pb intake were vegetables (25.4%), cereals (24.2%), and milk and dairy products (12.9%), while sweets contributed 8.2% to the total EDI of 24.57 μg Pb/person/day, a value lower than the reference JEFCA value for Pb. In another survey conducted in Riyadh, Nasser [51] measured levels of several heavy metals (Cd, Cu, Fe, Ni, Pb, and Zn), as well as fungal and microbial contents, in 13 samples of canned meats, primarily chicken and beef. Regarding the non-essential elements (Cd and Pb), Cd levels ranged between 0.16 and 0.62 μg/g, with four samples exceeding the maximum permissible level. Pb levels ranged from 0.27 to 1.1 μg/g, with all samples exceeding the maximum permissible level, and Ni was detected in three samples (all above the maximum permissible level for this element). The author highlighted the relevance of these results to prevent health risks to consumers, as canned meats are easily accessible and frequently consumed in the country. More recently, Korish and Attia [52] determined the levels of As, Cd, Co, Cr, Cu, Fe, Mn, Ni, Pb, Se, and Zn in samples of meat (fresh and frozen broilers), meat products (burgers and frankfurters), and chicken liver from markets in Jeddah. For the potentially most toxic elements, As was not detected in any sample, while Cd and Pb were not detected in broiler meat. However, both Cd and Pb were found in meat products (burger: 0.433 and 16.69 μg/g for Cd and Pb, respectively) and liver (1.12 and 16.51 μg/g for Cd and Pb, respectively). In another survey, Aljazzar et al. [53] measured Cd and Pb levels in samples of bovine meat and edible offal (round, tongue, colon, lung, rumen, liver, and kidney) from cattle in Al-Ahsa. As expected, the lowest mean concentrations of Cd and Pb were found in muscle samples: 0.21 and 0.06 μg/g, respectively. In contrast, the highest mean levels of both metals were detected in kidney (Cd: 0.21 μg/g) and liver (Pb: 0.55 μg/g). The EDIs for adults ranged between 0.04 mg/kg body weight/day (colon) and 0.11 mg/kg body weight/day (muscle) for Pb, and between 0.04 mg/kg body weight/day (tongue) and 0.40 mg/kg body weight/day (muscle) for Cd. Risk assessment for adults showed hazard ratios (HR) and hazard index (HI) values <1, including the combined risk of Pb and Cd for HI.

### 3.8. Other Asian Countries

In Taiwan, Chen et al. [54] measured the concentrations of As, Cd, Co, Mn, Mo, Pb, Se, and Sb in samples of livestock meat (beef, mutton, and pork) and poultry (chicken, duck, and goose). The ranges (μg/g) of the analyzed metals/metalloids were as follows: 0.005–0.035 (As), <0.002–0.003 (Cd), 0.002–0.033 (Co), 0.106–0.365 (Mn), 0.029–0.140 (Mo), 0.009–0.046 (Pb), 0.108–0.349 (Se), and <0.002–0.003 (Sb). In terms of the most toxic elements, Pb concentrations were higher in duck samples, while the highest As levels were found in chicken and pork samples. The results of this survey did not indicate any significant health risks for consumers. In Iraq, Ali et al. [55] determined the concentrations of Se and nine heavy metals (Cd, Co, Cr, Cu, Mn, Ni, Pb, Hg, and Zn) in chicken liver samples collected from markets in Erbil city, Kurdistan. For the three potentially most toxic elements, the mean concentrations (μg/g) were 0.07 ± 0.037 for Cd, 0.278 ± 0.10 for Pb, and 0.11 ± 0.083 for Hg. Lead, Hg, and Cu were the only analyzed elements that exceeded the permissible limits for poultry set by FAO/WHO. In Armenia, Pipoyan et al. [56] assessed dietary exposure to Ni for the adult population of Yerevan city, evaluating potential associated health risks using data from a 2018–2019 TDS. Composite meat samples (beef/veal, pork, sausages, chicken, and local foods like pelmeni and khinkali) included processed (grilled, boiled, and pan-fried) subsamples. The mean Ni concentrations (μg/g) in meat samples were as follows: beef/veal, 0.200; pork, 1.84; chicken, 0.192; sausages, 0.155; and pelmeni/khinkali, 0.196. The EDI of Ni for the population of Yerevan city was 0.147 μg/kg bw/day (specifically 0.157 and 0.142 μg/kg bw/day for females and males, respectively). The contribution of meat and meat products was relatively low compared to the main contributors: fruits and vegetables, and bread and flour-based products. In Pakistan, Mushtaq et al. [57] recently measured the levels of As, Cr, Co, Pb, Mn, and Hg in meat samples (including kidney and liver) from cattle, goats, and broilers collected in 20 different locations in Quetta (Balochistan). The highest concentrations of Cr, Pb, and Hg were found in liver samples. Most metal levels were within the normal ranges in the goat and cattle samples. In terms of EDIs, goat and cattle products were the main contributors to the intake of As, Co, Pb, and Hg, with Hg (and Cr) in adults exceeding the RfD (oral reference dose). Regarding the THQ, only As had a value >1.

Conversely, Hassan Emami et al. [62] recently published a review article summarizing and discussing available data on the levels of heavy metals in various red meat products consumed in the Middle East. Their review also examined the potential sources and pathways of exposure to these metals in the region. Unlike the present review, which focuses exclusively on Asian countries, Hassan Emami et al. [62] included studies not only from Asian countries (such as Iran, Iraq, Kuwait, Pakistan, and Saudi Arabia), but also from two African nations (Algeria and Egypt). The findings from most of the reviewed studies revealed that heavy metal concentrations in the analyzed meat samples exceeded the tolerable limits set by WHO/FAO and US EPA, indicating potential health risks.

## 4. Discussion

The aim of the present article was to review the scientific literature on the concentrations of As, Cd, Hg, Pb and other trace elements with potential health risks (mainly carcinogenic), such as Cr(VI) and Ni, in meat and meat products consumed in Asian countries. In addition, the potential health risks associated with human exposure to these elements through meat consumption, as well as their intake levels, have also been reviewed. According to documents in the PubMed and Scopus databases, Asia is the continent with the most information on this topic published in the current century, with China being the country with the largest number of available studies. Recently, Scutarasu and Trinca [63] published a comprehensive review on the occurrence of heavy metals across various food groups, including vegetables, fruits, milk and dairy products, meat and meat products, edible oils, and alcoholic beverages. Unlike the current study, their review encompassed not only toxic elements but also essential trace elements such as copper, iron, manganese, and zinc, among others. Furthermore, their analysis was global in scope, without geographic limitations to specific continents or countries. Based on the reviewed data, the authors identified As, Cd and Pb as the elements posing the highest potential health risks, with toxicity being directly linked to their accumulation in food products.

In specific regard to the current paper, it should be noted that the analytical methods used in the reviewed studies have not been discussed or questioned here. This is beyond the scope of the present review, as all studies included have already been published in international peer-reviewed journals. Therefore, it is assumed that the experimental methods employed were appropriate and acceptable according to the respective reviewers and editors of the papers. Thus, specific details about the analytical techniques are not included, as this information is typically provided in the Materials and Methods sections of the reviewed studies. For information purposes only, the techniques most used to determine the concentrations of the analyzed elements in meat samples were inductively coupled plasma mass spectrometry (ICP-MS) and atomic absorption spectrometry (AAS), including graphite furnace AAS. For mercury (Hg) and methylmercury (MeHg) analysis, cold vapor atomic absorption spectrometry (CV-AAS) was used in some studies as an alternative to ICP-MS, while hydride generation atomic absorption spectrometry (HG-AAS) was also applied.

The reviewed articles correspond to those from Asian countries, or regions within the same country, where results have been published in the current century. As expected, notable differences among the results of the reviewed studies were observed. These differences depend on various factors such as the animals’ species included in the respective studies, the consumed parts of the animals, the regions and countries where the samples were collected, and the dietary habits of the different population groups. Naturally, the highest concentrations were found in areas that are environmentally polluted by certain elements.

It is well established that humans are primarily exposed to chemical (both organic and inorganic) contaminants through their diet. For non-occupationally exposed populations, exposure to essential and non-essential trace elements mainly depends on dietary intake. Given the considerable variability in the studies reviewed here, along with the relatively small number of available scientific studies from Asia, it is difficult to draw definitive conclusions regarding the potential health risks of exposure to toxic trace elements through the consumption of meat and meat products. It is noteworthy that there are no publications on this topic in PubMed and Scopus for certain Asian countries, which does not imply that they have not been conducted. Simply, the results could have not been published in English in international scientific journals indexed in two of the most popular databases (PubMed and Scopus) used for searching information on topics like the current one. Considering the difficulties in comparing the results of the reviewed studies in terms of reported estimated daily intakes (EDIs) of the toxic trace elements, information from international organizations on the current tolerable daily/weekly/monthly intake levels for these elements is summarized next.

Regarding As, the IARC classified this element in Group 1 (carcinogenic to humans) in 2004. In 2009, the European Food Safety Authority (EFSA) concluded that the previously used Provisional Tolerable Weekly Intake (PTWI) of 15 μg/kg body weight (bw)/week was unsuitable, establishing a new Benchmark Dose Lower Confidence Limit (BMDL01) of 0.3–8 μg/kg bw/day for inorganic As (InAs). In 2010, based on new epidemiological data, the Joint FAO/WHO Expert Committee on Food Additives (JEFCA) set a safety limit of 3.0 µg/kg bw/day (range: 2–7 µg/kg bw/day, based on the range of estimated total dietary exposure). For inorganic As, the previous PTWI of 15 μg/kg bw/week (equivalent to 2.1 µg/kg bw/day) was deemed inappropriate. With respect to Cd, the IARC also classified this metal as a carcinogen to humans (Group 1). In 2005, JECFA established a PTWI of 7 μg Cd/kg bw/week. However, in 2009, EFSA re-evaluated the available data on Cd and set a new tolerable weekly intake (TWI) of 2.5 μg Cd/kg bw/week. In 2021, JECFA did not revise or modify the TWI for Cd. In relation to Hg and methylmercury (MeHg), in 2012, EFSA recommended a TWI of 4 μg/kg bw/week for inorganic Hg (InHg) and 1.3 μg/kg bw/week for MeHg. These same values were also recommended by JEFCA in 2011. Regarding Pb, the IARC classified it as a possible carcinogen to humans (Group 2B), and JECFA set a PTWI of 25 μg/kg bw/week. However, in 2010, EFSA concluded that there was insufficient evidence to support the health effects of Pb at the PTWI level of 25 μg/kg bw/week. As a result, EFSA recommended assessing Pb risk through Benchmark Dose Lower Confidence Limits (BMDLs) rather than tolerable intake values, due to the lack of sufficient evidence on health thresholds for Pb’s critical effects. In addition, EFSA published a scientific opinion on the risk to human health from Cr in food, mainly vegetables and bottled drinking water. A Tolerable Daily Intake (TDI) of 0.3 mg/kg bw/day was established for Cr(III), the essential nutrient and the main form of Cr in food. However, given that animal studies have shown that Cr(VI) may cause cancer, EFSA has not established a TDI for Cr(VI). For Ni, in 2020, EFSA updated its recommendations to avoid risks to human health from Ni in food and drinking water. Due to the application of the updated benchmark dose guidance, the TDI was increased from 2.8 μg Ni/kg bw/day to 13 μg Ni/kg bw/day.

## 5. Conclusions

Based on the results of the studies reviewed here, human exposure to As, Cd, Hg, and Pb, and other trace elements such as hexavalent chromium [Cr(VI)] and nickel (Ni), through regular or frequent consumption of meats, particularly poultry, pork, beef, and meat products (including offal), is unlikely to pose significant health risks to consumers. Notably, the contribution of Pb—one of the most analyzed and detected elements—to total dietary intake from meat and meat products is relatively low compared to other food groups. Environmental levels of Pb and Hg have declined significantly in recent decades, which is expected to further reduce the concentrations of these and other toxic elements in meat over time. To mitigate any potential health risks associated with exposure to toxic metals, metalloids, and organic contaminants through meat consumption, regular monitoring of these contaminants and assessments of human exposure are crucial. Interestingly, as observed with organic pollutants, meat and meat products are not among the primary dietary sources of toxic metals and metalloids for populations in Asian countries.

## Figures and Tables

**Table 1:** A summary of scientific studies conducted in China in the current century, focusing on the levels of metals and metalloids in meat and meat products, as well as their intake.

Region/Province/City	Metals and Metalloids Analyzed	Samples of Meat and Meat Products Analyzed	Intake of Metals Through Meat Consumption and Potential Risks for the Consumers *	Reference
Xiamen	Cd, Pb, Hg, As	Meat of chicken and pork, and offal	Pork meat was the main contributor to the EDI	Chen et al. [17]
Hong Kong	Al, Sb, Cd, Pb, MeHg, Ni, Sn, V	Data on the levels of meat/meat products were obtained from a Total Diet Study (TDS)	Meat and meat products was not the food group with the highest contribution to the daily intake of Cd, Pb and MeHg	Chen et al. [16]
Zheijang	Cd, Pb, Hg	Meat of pork	Among the samples of the food groups analyzed, the highest mean Pb level corresponded to meat of pork	Tang et al. [19]
Shenzhen	Cd, Hg, Pb, As, Cr, Cu, Fe, Zn, Mn, Mo, Ni, Co, Se and Ti	Pork, beef, duck and chicken. Also, animal viscera (chicken liver and gizzard; pig liver and kidney)	The total THQ values were >1 when the intakes of all analyzed elements in all meat products were considered.	Wu et al. [21]
Huainan	As, Cd, Co, Cr, Cu, Hg, Pb, Sb	Meat of beef, chicken, and duck	The EDIs for As, Cd, Hg and Pb, through the consumption of meats were: 0.041, 0.001, 0.001 and 0.176 μg/kg bw/day, respectively.	Cheng et al. [22]
Jiansgu Province	Cd	Data were obtained from the National Food Contamination Monitoring Program (2001–2009)	The highest Cd levels were found in kidney (followed by liver) of pig and sheep	Zhang et al. [23]
Wanshan, Guizhou Province	Hg, MeHg	Meat poultry (chicken, ducks and geese)	The highest concentrations of Hg and MeHg corresponded to the samples of liver, followed by blood	Yin et al. [24]
Guandong Province	As, Cd, Co, Cr, Cu, Mn, Ni, Pb, Se, Zn	Chicken (breast, drumstick, gizzard, heart, kidney and liver)	As, found at elevated levels in some samples, could mean health risks for the consumers	Hu et al. [25]
Chengdu	Mn, Zn, Cu, Sr, Cr, Ni, As, Cd, Pb, Co, Sb	Meat of pork, beef and poultry	In some meat samples, As, Cd and Cr levels were higher than the respective limit values	Wang et al. [26]
Beijing	Cr, Cd, Pb, As, Hg	Meats of mutton, beef, chicken and pork	The average concentrations of Cr, Cd, Pb, As, and Hg in meat samples were: 0.573, 0.015, 0.167, 0.053 and 0.018 μg/g, respectively.	Liang et al. [27]
Nandan County	Pb	Meats of pork, chicken and duck	The mean Pb concentration in meat samples was 3.22 (2.74–4.04) µg/g. The contribution to EDI of 381.8 µg Pb/day vs a total of 1400 µg Pb/day	Zhang et al. [23]
Heilongjiang, Jilin and Liaoning Provinces	Cd	Data were obtained from the 5th TDS. Meat and meat products included: pork, chicken breast, pig liver, sausage, roasted chicken, beef, pork skin aspic, lamb, dorking, braised spare, ribs in brown sauce, chicken liver, roasted duck and coagulated pig blood	Notable differences were found for the EDIs corresponding to the 3 Provinces. It, considering not only the levels of Cd in the analyzed samples, but also the different dietary habits	Wei and Cen [30]
Ten Chinese regions	Se and various associated metals	Se-rich food samples	The main dietary contributor of Se was meat. In that food group, the levels of Se were in the range 0.1728–0.2465 μg/g	Zhang et al. [31]
Nanjing	Cr, As, Cd, Hg, Pb	Pork muscle (shoulder, ham, loin, tenderloin and belly), and also offal (liver, kidney and intestine)	The EDIs (from pork consumption) were the following 3.00, 2.14, 1.00, 0.57 and 3.57 μg/kg bw/day for Cr, As, Cd, Hg, and Pb, respectively. All were below the recommended values.	Pei et al. [32]
Shenzen	Pb, Cd, Hg, As	Meats (not detailed in the paper)	The mean levels of Pb, Cd, Hg and As in meat samples were: 0.044, 0.0237, 0.0056, and 0.021 μg/g, respectively.	Wang et al. [33]
Zhejiang Province	As, Cd, Cr, Cu, Hg, Ni, Pb	Fresh meat of pork, beef, mutton, chicken and duck	The exposure assessment suggested relatively low health risks for subjects consuming meats. Only low percentages (0.09% for As, 0.19% for Hg, and 0.94% for Pb) exceeded the MACs set by the Chinese legislation	Han et al. [34]
Northern Ningxia	Al, As, Cr, Cd, Cu, Ni, Pb, Zn	Meats of pork, beef and mutton	The average concentrations (μg/g) of the most potentially toxic elements were: As, 0.218; Cd, 0.000; Cr, 0.472; Ni, 0.000, and Pb, 0.078	Wang et al. [35]
Zheijang Province	Ni	Sausages, Chinese bacon, and other meat products	The median level of Ni was 0.258 μg/g, for the set of analyzed samples, being the EDI at acceptable values	Han et al. [36]
Shandong Province	Cu, Cr, V, Ni, As, Se, Sn, Cd, Pb, Sb, Mn, Ba, Hg	Livestock meat and offal; poultry meat and offal	For the 13 analyzed elements, the total mean concentrations were 1.56 and 39.8 μg/g, in samples of meat and offal, respectively.	Qin et al. [37]
Chengdu	Cr, Cd, Cu, Zn, Ni, Pb	Pork, beef and chicken, as well as gizzard, heart, intestine, liver, kidney, tripe and brain of pig. Also duck intestines and aorta, rumen and omasum of bovine.	The only trace metal showing the highest concentration in meat and meat products was Zn. The rest of analyzed elements showed their respective highest levels in other food groups.	Zeng et al. [38]

* The intakes of the measured toxic elements through consumption of meat and meat products were only estimated in some studies. The current safety limits—set by international organizations—for the toxic elements As, Cd, Hg and Pb, are the following: As, 3.0 µg/kg bw/day (range: 2–7 µg/kg bw/day); Cd, 2.5 μg/kg bw/week; Hg, 4 μg/kg bw/week for inorganic Hg, and 1.3 μg/kg bw/week for MeHg, and Pb (not currently set; previously, 25 μg/kg bw/week.

**Table 2:** A summary of scientific studies conducted in Asian countries (excluding China) in the current century, focusing on the levels of metals and metalloids in meat and meat products, as well as their intake.

Country/Province/City	Metals and Metalloids Analyzed	Samples of Meat and Meat Products Analyzed	Intake of Metals Through Meat Consumption and Potential Risks for the Consumers **	Reference
Bangladesh, Dhaka	Pb, Cd, Cr, As, Hg, Mn, Fe and Zn	chicken meat (broiler, local, and sonali)	Only Pb levels exceeded the MAC. The THQ and the TTHQ for combined elements, and also the carcinogenic risks were below the respective allowable benchmarks.	Ullah et al. [39]
Bangladesh, Dhaka	Pb, Cd, Fe, Cr, Cu and Zn	chicken (muscle, liver, gizzard, heart, kidney, and brain)	The THQ and TTHQ values were found at <1 (no carcinogenic risks for the consumers), while the carcinogenic risks were within acceptable limits.	Hossain et al. [40]
Bangladesh, Dhaka, Chattogram, Gazipur, Rajshahi and Barisal	As, Cd and Pb	broiler chickens (bones, meat, and combinations of liver, kidney and gizzard)	While the concentrations of As and Cd were below the respective MRL, it was exceeded by Pb.	Bokthiar et al. [41]
Bangladesh, Noakhali	Cd, Cr, Pb, Ni, Fe, and Cu	samples of meat, brain and liver of poultry, somali chicken, cow, quail, duck, pigeon and goat	Considerable differences in metal levels were observed depending on the specific element, animal species, and tissue. Overexposure to Cd through meat consumption could increase the cancer risks, especially in children	Chowdhury and Alam [42]
India, Tamil Nadu (5 major metropolitan cities)	As, Cd, Hg and Pb	broiler chickens (breast, liver, neck, and kidney)	Although the levels of these elements exceeded MRL in some samples, the exposure to As, Cd, Hg, and Pb from chicken consumption was within acceptable limits.	Mathaiyan et al. [43]
India, Tamil Nadu (3 districts)	Cd, Pb and Zn	chickens (blood, intestine, breast, liver, and gizzard)	Blood samples from chickens in the 3 districts could pose potential risks to consumers. Liver samples from chickens in Coimbatore also exceeded the safety limits.	Pappuswamy et al. [44]
Thailand, Bangkok	As and Cd	poultry and pig (meat, liver and kidneys)	Consumption of these meats by children could mean high weekly and monthly intakes of As and Cd	Nookabkaew et al. [45]
Thailand, various regions	Pb, Cd, Co and Cr	duck meat	72% of the Cd levels and 66.7% of the Pb levels exceeded the standards set by FAO/WHO. However, the TTHQs for the 4 metals were below 1	Aendo et al. [46]
Iran, Teheran	Cd and Pb	cooked beef sausages (German, cocktail, hot dog, Lyoner, dry, and jambon)	The mean levels of Pb and Cd across all samples were 53.5 and 5.7 μg/g, respectively. The EDIs of Pb and Cd were 1.47 and 0.16 μg/day, respectively, both considered acceptable for consumers.	Abedi et al. [47]
Iran, Kurdistan Province, Sananday City	As, Se, Pb, Cd, Zn, Ni, Cu, Cr, and Co	beef, turkey, sheep, and ostrich	The THQ for Pb ranged from 0.0069 (ostrich) to 0.769 (beef), for Cd from 0.121 (ostrich) to 3.283 (beef), and for As from 0.057 (turkey) to 0.393 (beef). For Cd, the concentrations in beef (and sheep) were at a warning level.	Raeeszadeh et al. [48]
South Korea	As, Cd, Hg, and Pb	dishes already prepared to eat, including meat and meat products	The EDIs were 38.5, 14.3, 24.4, and 1.61 μg/person (based on a 55 kg body weight) for As, Cd, Pb, and Hg, respectively. All values were within safe limits.	Lee et al. [49]
Saudi Arabia, Riyadh City	Pb	various food groups. Meats were represented by chicken	The mean Pb concentration was 0.026 ± 0.018 μg/g, being under tolerable limits	Othman [50]
Saudi Arabia, Riyadh City	Cd, Cu, Fe, Ni, Pb, and Zn	canned meats, mainly chicken and beef	Regarding the toxic metals, Cd levels ranged between 0.16 and 0.62 μg/g, with 4 samples exceeding the maximum permissible level. Pb levels ranged from 0.27 to 1.1 μg/g, with all samples exceeding the maximum permissible level.	Nasser [51]
Saudi Arabia, Jeddah	As, Cd, Co, Cr, Cu, Fe, Mn, Ni, Pb, Se, and Zn	fresh and frozen broilers, meat products (burgers and frankfurters), and chicken livers	As was not detected in any sample. However Cd and Pb were found in meat products: burger, 0.433 and 16.69 μg/g, for Cd and Pb, respectively, but both metals were not detected in broiler meat.	Korish and Attia [52]
Saudi Arabia, Al-Ahsa	Cd and Pb	bovine meat and edible offal (round, tongue, colon, lung, rumen, liver, and kidney) from cattle	The EDIs for adults ranged between 0.04 (colon) and 0.11 mg/kg body weight/day (muscle) for Pb, and between 0.04 (tongue) and 0.40 mg/kg body weight/day (muscle) for Cd. Risk assessment for adults showed hazard ratios (HR) and hazard index (HI) values <1.	Aljazzar et al. [53]
Taiwan	As, Cd, Co, Mn, Mo, Pb, Se, and Sb	livestock meat (beef, mutton, and pork) and poultry (chicken, duck, and goose)	The ranges (μg/g) of the most toxic elements were: 0.005–0.035 (As), <0.002–0.003 (Cd), and 0.009–0.046 (Pb). The results of did not suggest significant health risks for the consumers.	Chen et al. [54]
Iraq, Kurdistan, Erbil City	Se, Cd, Co, Cr, Cu, Mn, Ni, Pb, Hg, and Zn	chicken liver	The mean concentrations (μg/g) of Cd, Pb and Hg were 0.07 ± 0.037, 0.278 ± 0.10, and 0.11 ± 0.083, respectively. Pb and Hg (also Cu) were the only elements exceeding the permissible limits for poultry	Ali et al. [55]
Armenia, Yerevan City	Ni	composite samples of beef/veal, pork, sausages, chicken, and local foods like pelmeni and khinkali. Processed (grilled, boiled, and pan-fried) subsamples were analyzed.	The EDI of Ni was 0.147 μg/kg bw/day (specifically 0.157 and 0.142 μg/kg bw/day for females and males, respectively).	Pipoyan et al. [56]
Pakistan, Balochistan, Quetta	As, Cr, Co, Pb, Mn, and Hg	meats (including kidney and liver) of cattle, goats, and broilers	The highest concentrations of Cr, Pb, and Hg were found in liver samples. For EDIs, goat and cattle were the main contributors to the intake of As, Co, Pb, and Hg, with Hg (and Cr) in adults exceeding the RfD. Only As had a THQ value >1.	Mushtaq et al. [57]

** The intakes of the measured toxic elements through consumption of meat and meat products were only estimated in some studies. The current safety limits, set by international organizations, for the toxic elements As, Cd, Hg and Pb, are the following: As, 3.0 µg/kg bw/day (range: 2–7 µg/kg bw/day); Cd, 2.5 μg/kg bw/week; Hg, 4 μg/kg bw/week for inorganic Hg, and 1.3 μg/kg bw/week for MeHg, and Pb (not currently set; previously, 25 μg/kg bw/week.

## Data Availability

No new data were created or analyzed in this study. Data sharing is not applicable to this article.
